# Applying the Delphi Approach to Prioritize Social Factors Affecting the Development of Children Under Six Years

**DOI:** 10.1186/s12889-023-16521-x

**Published:** 2023-08-29

**Authors:** Mitra Sadrkhanlou, Ali Maher, Khalil Alimohammadzadeh, Mehrnoosh Jafari, Mohammadkarim Bahadori

**Affiliations:** 1 Department of Health Services Management, School of Management, North Tehran Branch, Islamic Azad University, Tehran, Iran; 2 Department of Health Policy, Economics and Management and Medical Education, Shahid Beheshti University of Medical Sciences, Tehran, Iran; 3 Department of Health Services Management, North Tehran Branch, Islamic Azad University. Economics Policy Research Center, Tehran Medical Sciences, Islamic Azad University, Tehran, Iran; 4 Health Management Research Center, Baqiyatallah University of Medical Sciences, Tehran, Iran

**Keywords:** Child development, Inequity, Delphi approach, Social determinants of health

## Abstract

**Background:**

Social determinants of health have a key role in the growth and development of children, particularly in early childhood which is mentioned from infancy to the age of six years old. These factors might cause disparities in living conditions and consequently bring about inequities regarding different aspects of development such as emotional, psychological, social, psychological, and intellectual. This research aimed to provide a model for prioritizing social factors affecting the development of children under six years.

**Methods:**

We used quantitative–qualitative (mixed) method to perform data analysis. The statistical population included 12 medical experts and professionals in the field of children’s development and social determinants of health that were selected using the snowball method. In the quantitative section, a Delphi technique was applied to screen the extracted indicators. Then through applying a decision-making trial and evaluation laboratory (DEMATEL) method, the cause-and-effect interactions among main social determinants were identified. To analyze data, super decision software was used.

**Results:**

According to literature review and the results obtained from focus group discussions, five dimensions including individual factors, family factors, environmental factors, governance, and global factors were identified. Based on the study findings, the criterion of “family factors” was mentioned as the most important priority affecting childhood development. Furthermore, the sub-criterion of “International Programs and Policies” received the greatest priority among other sub-criteria with a profound impact on children's healthy growth and development.

**Conclusion:**

Despite the current knowledge about social determinants of health, it is required to identify the most influential socioeconomic factors on childhood development. In such a manner, political strategies for improving the health condition of children can be implemented based on scientific evidence. Due to the crucial role of family factors, environmental factors and other socio-economic conditions, health policy makers and public health practitioners should be informed of the importance of these factors in shaping the health condition of children.

**Supplementary Information:**

The online version contains supplementary material available at 10.1186/s12889-023-16521-x.

## Introduction

Sustainable development goals put emphasis on children’s health, demanding that each child has an equal right to survive, grow and suitably develop [1]. Paying attention to children's health is crucial as it impacts both on the health of future generations, the performance of communities and the issues facing the future of healthcare [2]. As early development of children begins before birth and keeps on until the first eight years of life, this period is regarded as the most important phase of life to invest in the development of human capital [3].

In every community, socioeconomic differences contribute to inequities in early childhood [2, 4, 5]. Whereby, children who live in socio-economically deprived conditions experience poor health compared to those living in more privileged areas. These socioeconomic inequalities are avoidable depending on the effectiveness of policy options implemented by governments. Furthermore, early development of children is significantly affected by the quality of the environment they live, the quality of parental care they receive, and the extent of support they obtain from their community [6]. In fact, the combination of biological and nurture factors is known as child development basics and constitutes the social determinants of health (SDH) with significant impact on early child development. Thus, a government which takes an active role in improving population health should address social determinants of health as key principles for improving more equitable health outcomes for communities [7]. Among factors affecting child development, SDH such as food, social class, employment, work condition, social support, addiction, social deprivation, stress, transportation, urbanization, and world migration significantly influence childhood growth and development [8, 9]. The World Health Organization (WHO) defines SDH as “conditions in which people are born, live, work, and age that affect people's health, well-being, and quality of life” [10]. According to Sustainable Development Goals (SDGs), there is an urgent need to end poverty and inequity and guarantee that all population can appropriately benefit from health, justice and well-being [11]. To do so, policy makers should mainly focus on three key domains of SDGs including economic, social and environmental factors. Several studies have revealed the advantages of early interventions from different individual, family, economic, and social aspects to resolve children’s developmental disorders in a timely manner [12–15].

Although SDH affects all age groups, its impact is more evident in children under six years old as the most vulnerable group of people toward health risks and environmental conditions. Experiences of children during this time period have a longstanding effect on their health and well-being aspects [2]. Literature validated these conclusions and explained that any distraction in this period brings about adverse health consequences among adults. Thus, in case of prompt action, favorable outcomes would be promoted and the influence of undesirable childhood experiences would be minimized in an effective manner [3]. It is estimated that more than 200 million children under the age of 6 fail to reach their full potential development in developing countries [16]. This figure shows a significant difference across countries. According to statistics, approximately 15–20% of children in the United States, 15% in Jamaica, 8% in Bangladesh, 15% in Pakistan, and 18.5–22% had some types of developmental disabilities [17–21].

Based on Bronfenbrenner's theory, child development is affected by numerous environmental layers among which family factors play an important role [22]. Accordingly, the conceptual framework of SDH introduced by WHO Commission on Social Determinants of Health (CSDH) has defined the relationship between different environmental layers and its impact on health. Variations in different developmental factors including social, psychological, emotional, and physical aspects can cause inequity in children’s health condition [3]. Such inequities particularly in the first 8 years of life are worrying as they paralyze the growth and development of some important skills and capabilities among individuals [23]. Therefore, it is recommended to determine key social determinants of health that are contributed to childhood well-being and their future health condition as a critical step in the pathway of gaining a comprehensive understanding about main influencing factors on children's development [24]. In sum, identifying the contributing factors and trying to reduce inequities through applying practical policies and practices is essential to consider the main important social determinants of child development based on a conceptual framework of the WHO Commission on Social Determinants of Health.

## Materials and methods

### Study design

This was a descriptive, cross-sectional study conducted in 2021 with a mixed-method explanatory design including a survey and focus group interviews. The study aim was to identify, prioritize and obtain the experts’ consensus about the most important and influential social determinants of health on childhood development through a modified Delphi approach. This approach enables an active effort to direct a group of experts in reaching consensus. For this purpose, an initial systematic search of the literature in the study area constituted a basis for Delphi rounds. Then, after each round a number of items that generated consensus were dropped for the following rounds and the panelists continued to discuss about the remaining factors [25].

### Participants

Snowball sampling was used to select a purposeful group of academic members and experts having knowledge of childhood development, and social determinants of health. When finding participants is a challenging issue, the use of snowball sampling helps scholars conduct studies straightforwardly specially in case of social research where it is necessary to find as many experts as possible to gather information about a certain subject [26]. Accordingly, 12 panelists were chosen to participate in the study and were organized in a focus group discussion (FGD) and Delphi-TOPSIS decision making process which was large enough to allow for variety of perspectives.

### Study phases

#### Phase 1- literature review

First to identify social determinants of health, a literature review was done through reviewing scientific databases of Web of Science, Scopus, Google Scholar, and Pub Med using keywords of child development, prioritization, and social determinants of health in an English language in the time period 1990–2020. As our research question is very specific, it is not required to collect data beyond the last 30 years. This time limit allowed us to include every possible information regarding the topic and collect as many studies as possible. As a result, all social determinants of health were extracted among which those better suited for childhood development were selected. In addition to literature review, the interview method was used to complete information about social determinants of health using focus group discussions. Accordingly, 12 panelists were chosen to participate in the study which was large enough to allow for variety of perspectives. The day began with an explanation about the project, and then study experts were organized in a focus group discussion (FGD). The FGD was held for two hours in March 2021 and facilitated by a research member. After obtaining the initial list of determinants, study experts were asked to review their earlier replies and revise them as necessary. Afterward replication, transparency, and applicability of factors were assessed by two research members in order to apply any necessary omission, revision or rewording in order to comply with study objectives.

#### Phase 2- Applying a Delphi questionnaire

By extracting and categorizing the indicators from the interview phase, a Delphi questionnaire was distributed among study experts and asked them to give a score between 1 to 10 based on the importance of each criterion. Based on the literature, if the average of experts’ opinions was lower than 7, that factor was removed, and if it obtained a score higher than 7, it was transferred to the next round of Delphi. This process continued until all indicators remained in the final round [27]. Ultimately, through analyzing experts’ opinions, 33 social determinants of health criteria in five categories were identified. Once the list of factors was finalized, a two-section questionnaire including one part gathering data on demographic characteristics and the second containing 39 questions with 5-point Likert scaling system was designed.

#### Phase 3- Applying a decision-making trial and evaluation laboratory (DEMATEL) method

DEMATEL method was used to identify the cause-and-effect interaction among main determinants through a causal diagram [28, 29]. In fact, to choose the ranking of strategies to improve the condition of children's development from the social perspective, experts were asked to determine the degree of direct impact between each pair of factors in form of pair-wise influence matrix X = (x_ij_) n × n, where n is the number of factor and xij shows the degree, to which i^th^ factor influences j^th^ factor. Five-point scale was used to determine the degree of influence from 0 (no influence) to 4 (very high influence) [30]. Problem solving by TOPSIS method was done in seven steps including: drawing the fuzzy adaptive matrix of experts’ viewpoint about the importance of each dimensions; converting the fuzzy decision matrix of viewpoints to a fuzzy unified matrix; creating a fuzzy weighted unified scale matrix; defining a fuzzy positive and fuzzy negative ideal for the criteria; calculating the sum of distances belonging to each criterion from positive and negative ideals; and ranking the solutions based on the descending order [31, 32].

### Statistical analysis

We sorted the research indicators using MAXQDA software and compiled the Delphi questionnaire. Then, we evaluated the internal associations between the main criteria using DEMATEL and assessed the sub-criteria of the determinants using the pair-wise comparison method. In order to calculate the reliability of obtained data from the Delphi approach, Kendall agreement coefficient was used. In this study, super decision software was used to analyze data and ethical considerations such as obtaining informed consent, preserving identity information of the participants and observing trustworthiness in implementing the content of interviews were observed.

### Ethical considerations

This study received approval from the Ethical Committee of the Islamic Azad University –Urmia Branch (IR.IAU.RECURMIA.REC1399.004).

## Results

Study findings revealed that most of the participants (83.3%) were female and the same percentage had PhD degree with an average age of 50.58 years old. As shown in Table 1, after applying a Delphi method, 33 social determinants of health in five categories including individual, family, environmental, governance and global factors were identified.

**Table 1:** Criteria and sub-criteria of social determinants of health

Dimension	Definition	Sub-dimension
Individual factors	order of birth among siblings	Birth rank
	The birth of more than one baby from a single pregnancy	Multiple births
	The health of women during pregnancy, childbirth and the postnatal period	Physical wellbeing during the child birth
	The cognition, communication, vision, hearing, social interaction, emotional response, adaptive behavior and physical condition of a child Experience with peers as an important developmental context for children through which they acquire a wide range of skills, attitudes, and behaviors	The status of childhood development Peer interactions, relationships and supportive groups
	Signs of health and normal function of the body system	Physical health status of the newborn
	Making sure that children eat healthy foods to grow and develop normally	Nutrition
Family factors	The extent to which a person has knowledge and skills	Parent's education and literacy level
	Level of understanding the parental roles	Parent's awareness level
	Parental employment status	Parent's employment and income status
	The state of having insufficient material possessions or little income	Poverty
	The degree of having money, property, or valuable possessions	Family wealth
	Any sort of pattern or dynamic that disrupts the household or family at large	Family Problems
	The state of physical, mental and social well-being of the mother	Mental and physical health of parents
	Bringing up a child or children without a partner	Being single parent
	The legal ending of a marriage	Divorce
	Natural birth, scheduled cesarean, unplanned cesarean, and vaginal birth after C-Section	Types of delivery
	The way each family member thinks, feels, and acts on a daily basis	Culture and values of the family
	Extent to which the ideal conditions for living are met	Level of technology
Environmental factors	Residential wellbeing and perceived safety in the living place	The security, safety, and health of the place of residence
	Facilities used for educational purposes	Educational facilities
	The physical conditions of a house, its surrounding and community	Quality of housing and living environment
	The amount of access to safe drinking water	Access to safe water and sanitation
	The facilities that are necessary for the well-being of residents	Welfare facilities of the place of residence
	Live permanently in a foreign country	Immigration
Governance factors	The perception of having assistance from other people	Social Support
	The availability of places that provide health care services	Healthcare facilities
	The attempts of governments to guide communities in attaining health	Performance and quality of educational, health, and social systems
	The measure of how social services are available and meet the residents’ expectations properly	Availability of services and their quality
	A platform for dissemination of information and a platform for interacting with one another	Media and social networks
Global factors	Global public policies and programs to promote the condition of SDH	International Programs and Policies
	Factors that affect the climate of a place	Climatic factors

To determine the degree of consensus among experts while using the Delphi method, Kendall rank correlation coefficient has been calculated. Table 2 depicts the value of Kendall rank correlation coefficient for two stages of the Delphi process. The Kendall rank coefficient was 0.315 in the first stage and 0.411 in the second stage showing a suitable consensus among experts.

**Table 2:** Delphi Kendall agreement coefficient of sub-criteria

	Number of indicators	Number of experts	Kendall coefficient	Consensus
First Round	39	12	0.315	weak
Second Round	33	12	0.711	strong

The content validity index (CVI) showed that all sub-dimensions had a CVI higher than 0.8; thus were mentioned as appropriate to be included in the final questionnaire. Table 3 demonstrates the calculated CVI values for each of the sub-indicators

To determine the causal relationships between dimensions and create the network structure using DEMATEL model, the initial relationship matrix for criteria was formed on the basis of experts’ opinion, which is shown in Table 3.

**Table 3:** Calculated CVI for questionnaire indicators

Criteria	Sub-criteria	CVI	Results
Individual Factors Related to the Child	Birth Order	0.83	Accepted
	Multiples	1.00	Accepted
	Physical Condition at Birth	1.00	Accepted
	Child Development Status	0.83	Accepted
	Interactions and Partnerships with Peers and the Environment	1.00	Accepted
	The State of Physical Health of the Child	1.00	Accepted
	Child Nutrition	0.83	Accepted
Family Factors	Parents' Education and Literacy	0.83	Accepted
	Parental Awareness	1.00	Accepted
	Parents' Employment Status and Family Income	0.83	Accepted
	Poverty	1.00	Accepted
	The Amount of Family Wealth	1.00	Accepted
	Family Problems	0.83	Accepted
	The level of mental-physical-spiritual health of parents	0.83	Accepted
	Being a Single Parent	1.00	Accepted
	Divorce	0.83	Accepted
	Type of Delivery	0.83	Accepted
	Family Culture and Values	0.83	Accepted
	Level of Technology	1.00	Accepted
Environmental Factors	The level of Security, Safety and Health of the Place of Residence	0.83	Accepted
	Impact of Educational Environment	0.83	Accepted
	Area and Place of Residence	1.00	Accepted
	Quality of Housing and Living Environment	0.83	Accepted
	Access to Safe Water and Sanitation	0.83	Accepted
	Living Amenities	1.00	Accepted
	Migration	1.00	Accepted
Governance Factors	Social Support	0.83	Accepted
	Health and Education	1.00	Accepted
	Performance and Quality of Educational, Health, and Community Systems	1.00	Accepted
	Availability of Services and their Quality	083	Accepted
	The Impact of Media and Social Networks	0.83	Accepted
Global Factors	International Programs and Policies	0.83	Accepted
	Climatic Factors	0.83	Accepted

Then, direct relationship matrix was normalized (Table 4) and used to obtain total influence matrix (Table 5). Using the geometric mean technique and normalization of obtained values, the eigenvector was calculated.

**Table 4:** Direct relation matrix: M

	C1	C2	C3	C4	C5
C1	0.0000	3.250	3.125	2.250	1.500
C2	2.275	0.000	3.250	2.275	1.625
C3	2.625	2.875	0.0000	3.125	2.125
C4	2.250	3.000	2.750	0.000	2.875
C5	2.250	2.625	2.000	3.000	0.000

C represents for criteria

**Table 5:** Normalized matrix: N

N	C1	C2	C3	C4	C5
C1	0.000	0.277	0.266	0.191	0.128
C2	0.234	0.000	0.277	0.234	0.138
C3	0.223	0.245	0.000	0.266	0.181
C4	0.191	0.255	0.234	0.000	0.245
C5	0191	0.223	0.170	0.255	0.000

**Fig. 1:**
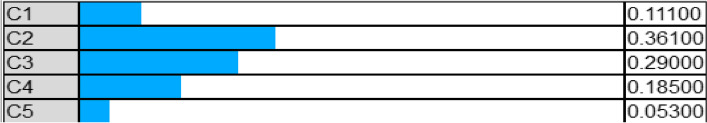
The Output of super design software

Figure 1 compares the importance of main study criteria. As it is observed in Fig. 1, family factors and environmental factors received the highest priority; while global factors were mentioned to have the least priority.

Next, sum of rows and columns was calculated from total influence matrix. The sum of rows and columns is represented by vector ‘R' and ‘D', respectively (Table 6).

**Table 6:** Total influence matrix: T

T	C1	C2	C3	C4	C5
C1	1.35	1.768	1.711	1.64	1.255
C2	1.567	1.583	1.748	1.699	1.289
C3	1.597	1.823	1.571	1.762	1.352
C4	1.585	1.839	0.234	1.563	1.404
C5	1.477	1.696	1.608	1.648	1.117

**Fig. 2:**
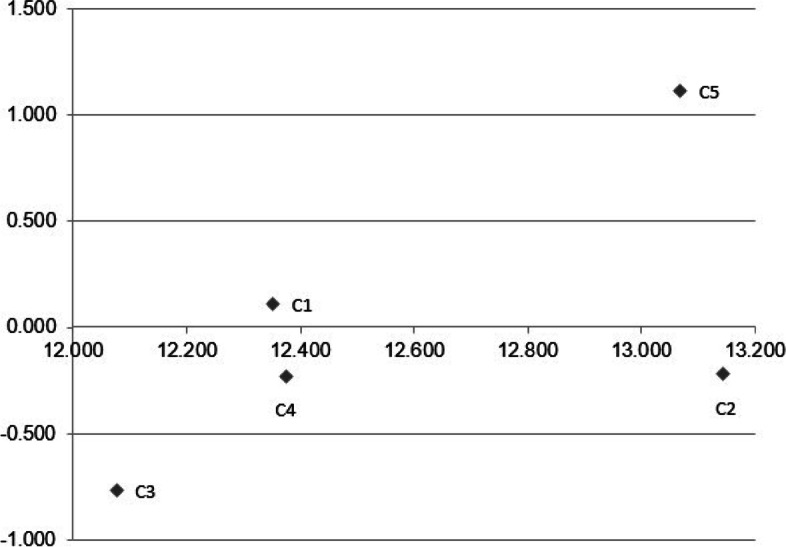
Causal influential diagram for the DEMATEL model

In Table 6, sum of the values of each row (D) indicates the effect of that criterion on other criteria. Accordingly, the most influential factor is governance. Furthermore, the sum of values regarding to each column (R) represents the extent that a definite factor is influenced by other criteria. As a result, family factors are mostly affected by other social determinants. In the last step of applying DEMATEL model, an influential graph was used to help decision makers find the most influencing social determinants of health. In Fig. 2, X-axis represents the values of R + D, and U-axis includes the values of R-D. The most influential factors are at the highest level (top) while the least affecting factors are at the bottom.

As shown in Table 7, family factors, environmental factors and governance fall in the category of effect group that are easily influenced by other factors and show the least impact. Such factors are the main issues and cannot change the system specifically.

**Table 7:** Sum of rows and columns of matrix T

Criterion	R	D	D-R	D + R
Individual factors related to the child	7.577	7.725	0.147	15.302
Family factors	8.709	7.886	-0.822	16.595
Environmental factors	8.408	8.105	-0.303	16.513
Governance factors	8.311	8.161	-0.149	16.472
Global factors	6.417	7.545	1.127	13.962

Finally, the study sub-criteria were compared in pairs. Related results are shown in Table 8. As it is shown, the highest priority was respectively given to international programs and policies, climate factors and availability of services and their quality.

**Table 8:** Priority ranking of sub-criteria

**Sub-criteria**	**Normal weight**	Ranking
International Programs and Policies	0.1166	1
Climatic factors	0.0831	2
Availability of services and their quality	0.0519	3
Nutrition	0.0502	4
Performance and quality of educational, health, and social systems	0.0485	5
Peer interactions, relationships and supportive groups	0.0438	6
Social support	0.0396	7
Educational environment	0.0377	8
Physical health status of child	0.034	9
Immigration	0.0329	10
Child Development	0.0318	11
Healthcare facilities	0.0317	12
Access to safe water and sanitation	0.0311	13
The security, safety, and health of the place of residence	0.0303	14
Media and social networks	0.0284	15
Quality of housing and living environment	0.0261	16
Welfare facilities of the place of residence	0.0245	17
Parent's employment and income	0.0244	18
Parent's awareness level	0.0215	19
The physical condition during the birth	0.0204	20
Place of residence	0.0177	21
Type of delivery	0.016	22
Culture and values of the family	0.016	23
Level of technology	0.016	24
Poverty	0.016	25
Family wealth	0.016	26
Family problems	0.016	27
Mental and physical health condition of parents	0.016	28
Single parenthood	0.016	29
Divorce	0.016	30
Childbirth rank of family	0.0106	31
Parent's education and literacy level	0.0104	32
Multiple birth	0.009	33

## Discussion

Study findings revealed that social determinants of health in five main domains of individual factors, family factors, environmental factors, governance and global factors can significantly affect early childhood development and how well they live throughout the years of adulthood. Identification and prioritization of these factors improves the ability of policy makers to make evidence-based decisions regarding the most influential factors in closing the health inequalities gap. Although some layers of influencing factors are simpler to be resolved than others, a multi-faceted approach toward social determinants of health is required to more appropriately respond to complex health issues [33, 34]. Among major criteria of the research, family factors got the most priority. In fact, a family can positively affect both physical and mental health condition of children through creating a supportive emotional and learning environment; and providing suitable access to educational, health and social services [34–36]. In the literature, different variables were mentioned to have influence on family function including parents’ educational levels, occupation and monthly income [37]. It has been emphasized that poverty can restrict access to proper nutrition, safe housing, and appropriate educational facilities [38, 39].

Social and environmental influences contribute between 45 and 60% of the variation in a community health status [40]. Some of the main environmental factors which endanger human health include climate change, air pollution, chemical pollution, infectious diseases, lack of access to health care services, poor sanitation and water quality. Vulnerable groups such as those living in hazardous climate-related conditions are confronting with the risk of extreme weather phenomena such as river flows, hurricane, strong winds, cold temperatures, floods, etc. which bring about adverse health effects for the population [41].

Among sub-criteria, international policies; climate factors; availability of services and their quality; nutrition; the quality of educational, Health and social systems; peer interactions and supportive group were regarded as six top priority determinants identified by study experts. The WHO Global Commission on the Social Determinants of Health (CSDH) emphasized that provision of universal access to healthcare services should be included in international programs and policies of worldwide countries. As a significant strategy the United Kingdom provided universal health coverage to resolve persistent inequalities in health between social groups [42]. However, experience has shown that improving the health condition of communities not only depends on reducing health inequalities but also needs a considerable improvement in the conditions in which individuals are born, live, and work [40]. To resolve the issue, a global action towards health is required in all policies. Following the WHO CSDH, the Rio Political Declaration on social determinants of health put an emphasis on global political commitment for the implementation of SDH approach to reduce health inequities [43]. Such a worldwide endeavor would help countries to put force for the development of national strategies to address the underlying determinants of health [42]. Since unequal opportunities in a community will have adverse effects on the development of children's capacities, governments have intervened in the operations of equitable policies for children from early childhood through prioritizing them in the welfare promoting programs, empowering families and expanding educational social support services for them to achieve developmental goals [3].

Climate factor had the second priority ranking among identified sub-criteria. In this regard, the social determinants of health department in the WHO European Region highlighted three main dimensions of physical environment affecting children’s health as key determinants of health inequalities [44]. These main aspects include poor housing, climate change and air pollution which provide unequal conditions as important factors in generating health inequities [45]. In recent years, climate change has worsened health condition of communities through extreme weather phenomena, reduced productivity and its effects on malnutrition, infectious disease transmission and forced migration [46]. These harmful outcomes will increase health inequities and lead to a rising community health problem [47]. Thus, in developing countries, clean water and sanitation; appropriate nutrition; immunizations and availability of educational, health and social support systems are key determinants of health. Inequity in children's access to nutrition, quality health services, and education in early childhood significantly decreases children's chances of achieving potential development [48]. Furthermore, reducing social capital and cohesion as well as lack of peer interactions and supportive groups bring about negative effects on children's development. Powers et al. emphasized that poor social support is associated with alcohol drinking and smoking during pregnancy and consequent maternal depression which potentially leads to a decreasing level of health condition among children [3]. This finding is in line with our study results. Cultural factors are other influential elements that affect the physical, mental and emotional development of children. These factors come out in the form of lack of awareness relating to parenting methods and the importance of early childhood development. Such misunderstandings can be resolved through increasing parents' awareness and delivering educational programs [3]. Therefore, the role of media and social networks in increasing the literacy level of parents and resolving existing misconceptions about nurturing methods should be properly considered [48].

## Conclusion

The importance of social determinants versus genetic factors is often discussed in various literatures. Despite the current knowledge about social determinants of health, it is required to identify the most influential socioeconomic factors on childhood development. In such a manner, political strategies for improving the health condition of children can be implemented based on scientific evidence. Due to a crucial role of family factors, environmental factors and other socio-economic conditions, health policy makers and public health practitioners should be informed of the importance of social factors and their power in shaping health.

### Practical implication of the study

Based on the study results, there is a necessity to provide a proper socio-economic, cultural and political environment that facilitates constructive interactions between children and their parents leading to positive learning experiences with sufficient support. To ensure that inequity in SDH is prohibited particularly during the early childhood years, it is recommended to work collectively and encourage researchers to investigate how different socio-economic, cultural and geographical conditions affect the health and well-being of children. In this manner, developing policy solutions would be much more plausible and practical.

### Supplementary Information

**Additional file 1.**

## Data Availability

The datasets used and/or analyzed during the current study are available from the corresponding author on reasonable request.
