# Supplementary material for: Applying the Delphi Approach to Prioritize Social Factors Affecting the Development of Children Under Six Years
Source: BMC Public Health. 2023 Aug 29;23:1659. doi: 10.1186/s12889-023-16521-x (PMC10466685; doi:10.1186/s12889-023-16521-x)
Supplement: Supplementary file 1 [file 12889_2023_16521_MOESM1_ESM.odt]

search databases

  - English databases including Web of Science, Scopus, Google Scholar, and Pub Med 

  - Persian databases including SID, MagIran

Analysing abstract

  - Filtering irrelevant papers

Full-text review

  - Extracting irrelevant papers 

Reference scanning 

  - Adding relevant papers 

Analysis of literature review

  - Extracting irrelevant papers

Supplementary file 1. Steps of Literature Review

	

Supplementary file 2. Data Extraction Form 

| Study characteristics | Descriptions as stated in paper |
| --- | --- |
| Study objective |  |
| Study design |  |
| Unit of allocation |  |
| Start date |  |
| End date |  |
| Duration of participation |  |
| Population |  |
| Setting |  |
| Inclusion criteria |  |
| Exclusion criteria |  |
| Intervention |  |
| Outcome |  |
| Summary of the results |  |
